# The usefulness of intraoperative neurological monitoring for esophageal cancer with double aortic arch; a case report

**DOI:** 10.1186/s12893-020-00751-6

**Published:** 2020-05-04

**Authors:** Shu Mushiake, Kohei Taniguchi, Sang-Woong Lee, Tetsunosuke Shimizu, Yoshiro Imai, Ryo Tanaka, Kotaro Honda, Keitaro Tashiro, Masaru Kawai, Kazuhisa Uchiyama

**Affiliations:** 1 Department of General and Gastroenterological Surgery, Osaka Medical College, 2-7 Daigaku-machi, Takatsuki, Osaka 569-8686 Japan; 2 Translational Research Program, Osaka Medical College, 2-7 Daigaku-machi, Takatsuki, Osaka 569-8686 Japan

**Keywords:** Double aortic arch, Intraoperative neurological monitoring system, Recurrent laryngeal nerve, Case report

## Abstract

**Background:**

Double aortic arch (DAA) is a congenital anomaly of the aorta. Esophageal cancer with DAA is rare, and consequently, the appropriate surgical approach has not been standardized. Herein, we report the utilization of intraoperative neurological monitoring (IONM) system to preserve the function of the recurrent laryngeal nerve.

**Case presentation:**

A 79-year-old man with esophageal cancer was diagnosed with DAA incidentally. The descending aorta was located on the right side of the thoracic vertebrae. Safe dissection of the mediastinal lymph nodes was difficult using the right transthoracic approach because of the anatomical abnormalities. During surgery, we used cervical mediastinoscopy combined with the IONM system to preserve the bilateral recurrent laryngeal nerves. Severe complications, including recurrent nerve palsy, were not observed postoperatively.

**Conclusion:**

IONM may be useful for evaluation of the function of the recurrent laryngeal nerve, and it would be suitable for atypical cases of esophageal cancer.

## Background

Double aortic arch (DAA) is a condition caused by congenital anomalies of the aorta and its bifurcations. DAA is a type of vascular ring of the aorta and a rare disease that accounts for 1–2% of congenital cardiovascular abnormalities [1]. Most DAA-patients present with specific symptoms, such as dyspnea and food dysphagia, and they are commonly treated at childhood. Based on this, adult diseases with DAA, including esophageal cancer, are extremely rare. Esophageal cancer with DAA presents an anatomical difficulty in surgical treatment. In esophageal cancer surgery, the preservation of the function of the recurrent laryngeal nerve is one of the important matters. Herein, we report an extremely rare case of an adult esophageal cancer with DAA and demonstrate the usefulness of intraoperative neurological monitoring (IONM) system for the preservation of recurrent laryngeal nerve function.

## Case presentation

**Fig. 1:**
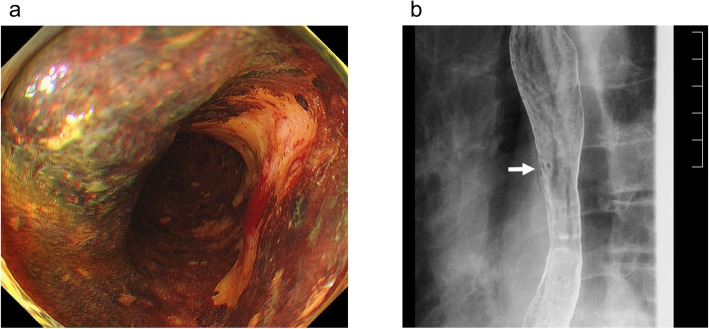
Preoperative imaging findings. **a** Esophagoscopy reveals a 0-IIc type tumor with unstained iodine occupying half the circumference of the esophageal wall. **b** Esophagogram shows irregular mucosa on the right side of the esophageal wall at the level of tracheal bifurcation (white arrow)

**Fig. 2:**
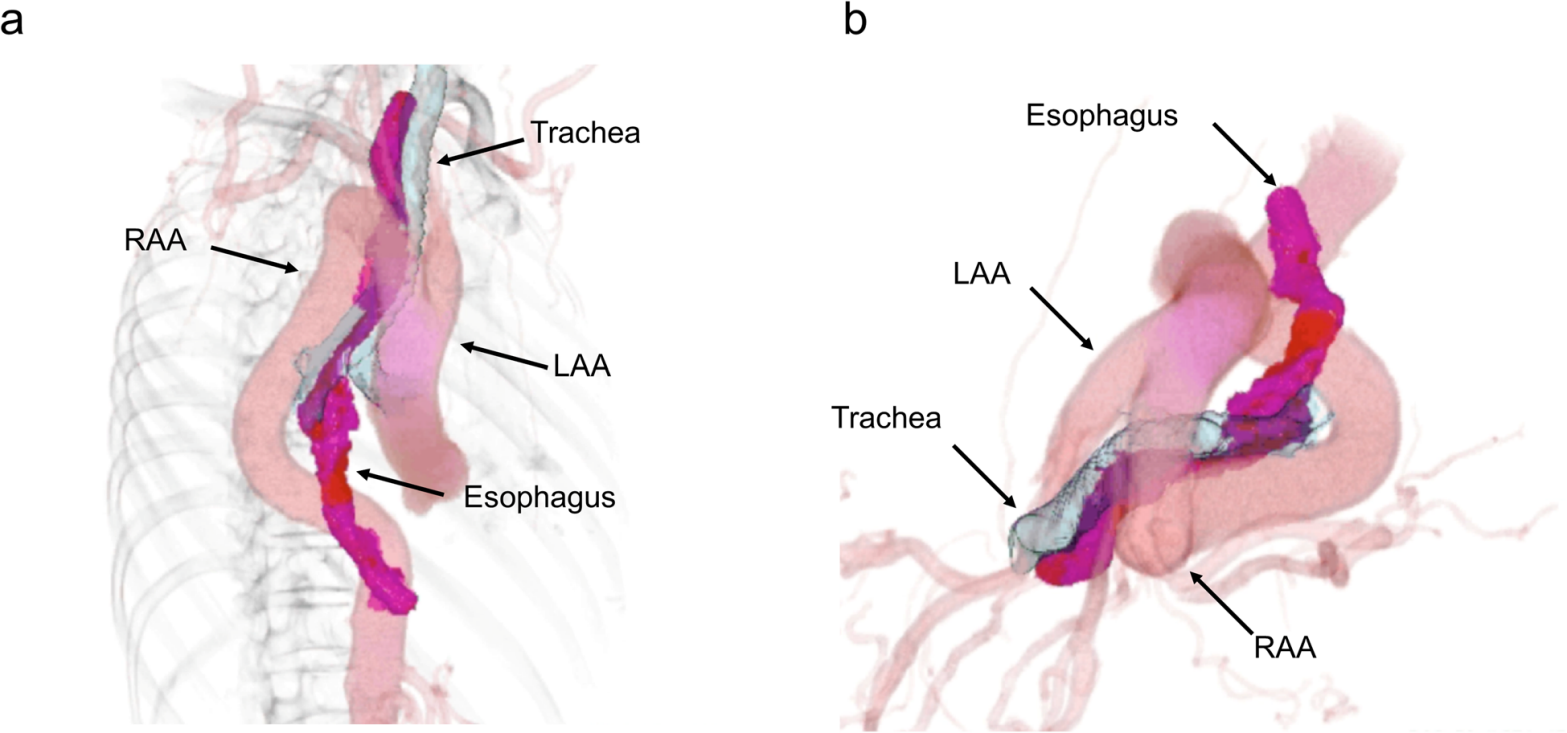
Aorta-related imaging findings**. a** Contrast-enhanced computed tomography (CT) shows a double aortic arch (DAA), and the descending aorta is located on the right side of the thoracic vertebrae. **b** The trachea and esophagus are encircled with the right aortic arch (RAA) and left aortic arch (LAA)

A 79-year-old man had anemia, and an esophageal tumor was detected via upper gastrointestinal endoscopy at a local hospital. He was referred to our department for surgical treatment of the esophageal tumor. Physical examinations, including heart and breathing sounds, were normal. The laboratory tests showed renal dysfunction (creatinine clearance: 37 mL/min/1.73 m^2^; normal range, 82–183 mL/min/1.73 m^2^). His carcinoembryonic antigen (CEA) level was normal, but his squamous cell carcinoma (SCC)-related antigen level was slightly elevated (1.8 ng/mL; normal range, < 1.5 ng/mL). Esophagoscopy revealed a 0-IIc type tumor with unstained iodine, occupying half the circumference of the esophageal wall in the middle thoracic esophagus area (Fig. 1a). Esophagogram showed irregular mucosa on the right side of the esophageal wall at the level of tracheal bifurcation (Fig. 1b). Histopathological examination of the biopsy samples showed SCC. Based on these findings, we concluded that the primary tumor was cT1b. DAA was detected incidentally on contrast-enhanced computed tomography (CT), and the descending aorta was located on the right side of the thoracic vertebrae (Fig. 2a). The trachea and thoracic esophagus were encircled by the right and left aortic arch (Fig. 2b). The right aortic arch (RAA) was larger than the left aortic arch (LAA); however, both aortic arches were patent. Based on these findings, the diagnosis was thoracic esophageal cancer (clinical stage I) with DAA (type IA; Edward’s classification). Neoadjuvant chemotherapy was not performed owing to renal dysfunction; thus, we decided to perform surgical treatment.

**Fig. 3:**
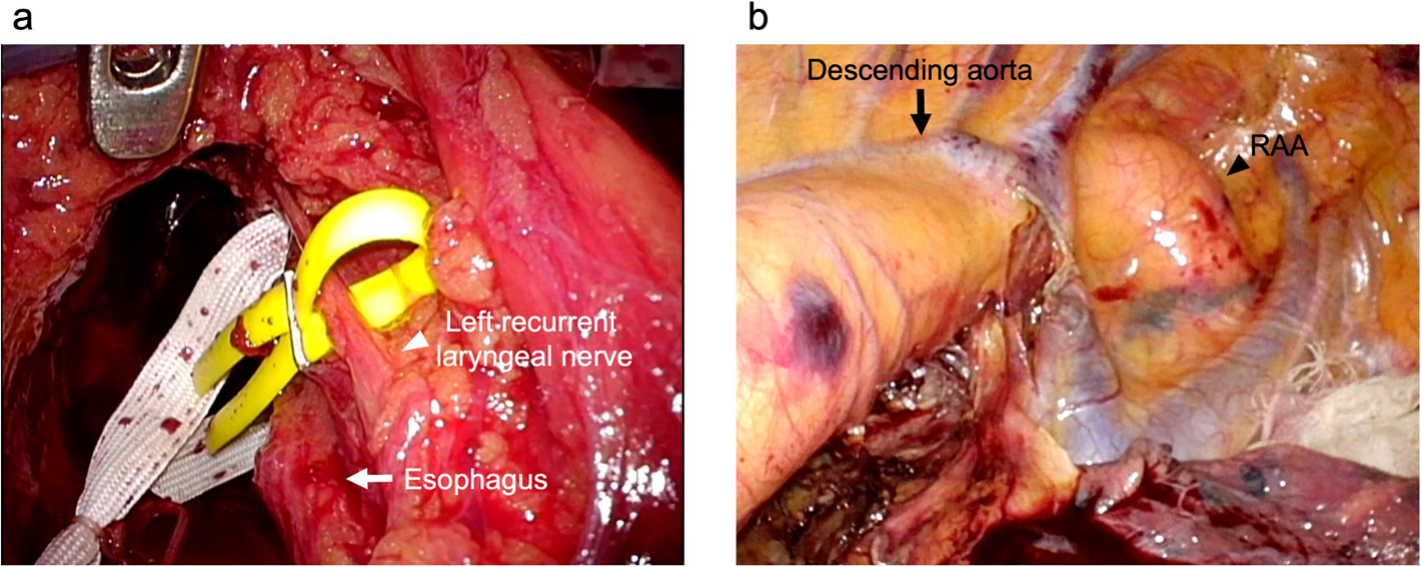
Intraoperative images**. a** Both recurrent laryngeal nerves are separated using yellow vessel tapes. The picture shows left recurrent laryngeal nerve with yellow vessel tapes (white arrowhead). The esophagus is indicated by the white arrow. **b** The descending aorta is seen meandering to the extreme right in the middle (black arrow) and lower mediastinum, and the right aortic arch (RAA) is located on the upper mediastinum (black arrowhead)

The operation consisted of three steps. The first step was the dissection of upper mediastinal lymph nodes and confirmation of the location of the bilateral recurrent laryngeal nerves using cervical mediastinoscopy with the patient in the supine position. We identified the vagus nerve leading to each recurrent laryngeal nerve at the upper mediastinum (Fig. 3a). We put the IONM (NIM-Response 3.0 SystemTM, Medtronic, Jacksonville, FL) monitor on both vagus nerves during the cervical approach with mediastinoscopy. During thoracoscopic surgery, we continuously ran the IONM to examine the response of the nerves. We stimulated both recurrent laryngeal nerves. We could confirm the response of the right side, but that of the left side could not be confirmed. The second step consisted of a detachment of the esophagus using a video-assisted right thoracic approach with the patient in the prone position. The aorta was meandering to the extreme right in the middle and lower mediastinum, and the RAA was located on the upper mediastinum (Fig. 3b). The degree of adhesion around the aortic arch was not so strong, and we easily remove it from the surrounding tissue. Subsequently, we could confirm that the right and left recurrent laryngeal nerves were recurring at the RAA and LAA, respectively. After careful dissection around both recurrent laryngeal nerves, the transection of the thoracic esophagus was performed. The last step was the laparoscopic creation of a gastric conduit, which was pulled up through a retrosternal route. Finally, cervical esophagogastric anastomosis was performed.

Macroscopic findings showed SCC within the submucosal invasion (350 μm) and no metastatic lymph nodes at any site. This case was classified as Mt, pT1b, N0, M0, pStage I. The patient developed aspiration pneumonia (Grade II by Clavien-Dindo classification), but hoarseness did not appear. The patient was transferred to another hospital for rehabilitation on postoperative day 36, and no recurrence was seen for 18 months after surgery.

## Discussion and conclusions

According to Stewart and Edward’s classification, our case of DAA was type IA (the type of patency of both sides). In this type, the LAA and RAA surround the trachea and esophagus [2]. Based on the normal embryology, the location of the recurrent laryngeal nerve is generally thought to be recurrent to the bilateral aortic arch, but this is not always expected in DAA cases [3, 4].

The IONM is a nerve stimulation monitor that uses a probe to apply electrical stimulation to nerves during surgery and analyzes myoelectric activity. It has been reported that IONM can prevent injury to the nerve during neck dissection [5]. We used the IONM system to confirm myoelectric activity continuously and preserve the bilateral recurrent laryngeal nerves because we could not predict the location of the bilateral recurrent laryngeal nerves due to embryological anomaly and also assumed that a non-recurrent laryngeal nerve might exist. To the best of our knowledge, although there are no reports regarding the existence of a non-recurrent laryngeal nerve in DAA cases, a non-recurrent laryngeal nerve is common in RAA cases [6]. Therefore, we considered the existence of a non-recurrent laryngeal nerve in this DAA case. However, in this case, no non-recurrent laryngeal nerve was found. Rather, we could confirm the location of the bilateral recurrent laryngeal nerves by using IONM. In particular, we were able to ascertain that the right recurrent laryngeal nerve was located and had recurred on the RAA. The validation of the function of the nerve and its location are important in each case, and IONM is a suitable device to confirm the location and function of the nerve during surgery in patients with anatomical abnormalities, as in the present case. However, the potency of the IONM for preserving nerve function remains controversial. Using IONM does not mean that nerve function can be preserved [7]. Furthermore, the additional time needed to set the device for the cervical approach can be considered a disadvantage of IONM.

A surgical approach should be considered carefully in cases of esophageal cancer with DAA. In this case, the bilateral recurrent laryngeal nerves had embryological abnormalities, and the descending aorta was meandering to the extreme right in the middle and lower mediastinum. Thus, we considered that dissection of the recurrent laryngeal nerve lymph nodes using a conventional right thoracic approach would be difficult. We selected IONM with cervical mediastinoscopy for dissection of the upper mediastinal lymph nodes with the patient in the supine position. We could observe the level of the lower edge of the aortic arch that could not be observed with a conventional approach. This is because we had thought that the adhesion around the esophagus was strong owing to contact stimulation with the DAA. We could dissect the upper mediastinal lymph nodes using IONM and examine the response of both recurrent laryngeal nerves. Furthermore, using cervical mediastinoscopy with the patient in the supine position, we could safely dissect and confirm both recurrent laryngeal nerves continuously during the cervical surgery. Esophagectomy for thoracic esophageal cancer is usually performed using right thoracotomy in consideration of the anatomical relationship between the esophagus and the descending aorta. The esophagus is located on the right side of the aortic arch in most normal cases. It has been reported that thoracotomy for surgical treatment in adult patients with DAA should be performed on the side with the more atrophic arch [8]. On the other hand, Fujiwara et al. reported that the surgical approach differed in each of the six cases of esophageal cancer with DAA [9]. In the present case, we selected right thoracotomy with the patient in the prone position because we judged that a more familiar method was suitable for this atypical case. Two major reconstruction routes are created after conventional esophagectomy - the retrosternal route and posterior mediastinal route. Each reconstruction route has some advantages and disadvantages; therefore, these two routes are used to similar extents in Japan. The posterior mediastinal route is better as it avoids compression of the reconstruction route by the sternoclavicular joint. In patients with DAA, there is an adhesion around the esophagus owing to long-term contact between the trachea and DAA. Hence, we judged that the retrosternal route was more suitable than the posterior mediastinal route. Accumulation of experience regarding reconstruction routes for esophageal cancer with DAA is required.

In conclusion, we experienced a case of esophageal cancer with DAA, and we performed esophagectomy without severe complications. Patients with DAA often have anatomical abnormalities, and the function of the bilateral recurrent laryngeal nerves differs in each patient with DAA. Cervical mediastinoscopy with IONM was effective during surgery to reduce the complications caused by damage to the recurrent laryngeal nerves. Thus, this method may assist in atypical cases of esophageal cancer.

## Data Availability

Not applicable.

## Abbreviations

CEA: Carcinoembryonic antigen
CT: Computed tomography
DAA: Double aortic arch
IONM: Intraoperative neurological monitoring
LAA: Left aortic arch
RAA: Right aortic arch
SCC: Squamous cell carcinoma
